# Crystal structure of UDP-glucose:anthocyanidin 3-*O*-glucosyltransferase from *Clitoria ternatea*

**DOI:** 10.1107/S0909049513020712

**Published:** 2013-09-29

**Authors:** Takeshi Hiromoto, Eijiro Honjo, Taro Tamada, Naonobu Noda, Kohei Kazuma, Masahiko Suzuki, Ryota Kuroki

**Affiliations:** a Quantum Beam Science Directorate, Japan Atomic Energy Agency, 2-4 Shirakata-Shirane, Tokai, Ibaraki 319-1195, Japan; b Institute of Floricultural Science, National Agriculture and Food Research Organization, 2-1 Fujimoto, Tsukuba, Ibaraki 305-8519, Japan; c Institute of Natural Medicine, University of Toyama, 2630 Sugitani, Toyama 930-0194, Japan; d Graduate School of Agriculture, Hokkaido University, Kita 9, Nishi 9, Kita-ku, Sapporo, Hokkaido 060-8589, Japan

**Keywords:** glucosylation, glucosyltransferase, anthocyanidin, crystal structure

## Abstract

Flowers of the butterfly pea (*Clitoria ternatea*) accumulate a group of polyacylated anthocyanins, named ternatins, in their petals. The first step in ternatin biosynthesis is the transfer of glucose from UDP-glucose to antho­cyanidins such as delphinidin, a reaction catalyzed in *C. ternatea* by UDP-glucose:anthocyanidin 3-*O*-glucosyltransferase (*Ct*3GT-A; AB185904). To elucidate the structure–function relationship of *Ct*3GT-A, recombinant *Ct*3GT-A was expressed in *Escherichia coli* and its tertiary structure was determined to 1.85 Å resolution by using X-ray crystallography. The structure of *Ct*3GT-A shows a common folding topology, the GT-B fold, comprised of two Rossmann-like β/α/β domains and a cleft located between the N- and C-domains containing two cavities that are used as binding sites for the donor (UDP-Glc) and acceptor substrates. By comparing the structure of *Ct*3GT-A with that of the flavonoid glycosyltransferase *Vv*GT1 from red grape (*Vitis vinifera*) in complex with UDP-2-deoxy-2-fluoro glucose and kaempferol, locations of the catalytic His-Asp dyad and the residues involved in recognizing UDP-2-deoxy-2-fluoro glucose were essentially identical in *Ct*3GT-A, but certain residues of *Vv*GT1 involved in binding kaempferol were found to be substituted in *Ct*3GT-A. These findings are important for understanding the differentiation of acceptor-substrate recognition in these two enzymes.

## Abstract 2

The anthocyanidin 3-*O*-glucosyltransferase from *Clitoria ternatea* (*Ct*3GT-A) was expressed in *Escherichia coli*, and the three-dimensional structure of *Ct*3GT-A was determined using X-ray crystallography. This report describes the architecture of *Ct*3GT-A, including the structures of the donor- and acceptor-binding sites.

## 1. Introduction

Many small lipophilic compounds in living cells are modified by glycosylation, a process that can regulate the bioactivity of those compounds, their intracellular localization and their metabolism (Lim & Bowles, 2004 ▶). One of the most significant, and representative, glycosylation reactions in plants is the formation of anthocyanins, a class of flavonoids. Anthocyanins are water-soluble compounds based on a tricyclic flavonoid core and are known to function as pigments involved in determining the color of flowers, leaves, seeds and fruits (Offen *et al.*, 2006 ▶; Tanaka *et al.*, 2008 ▶).

The blue flower pigmentation of *Clitoria ternatea* results from the accumulation in the petal of polyacylated anthocyanins referred to as ternatins (Honda & Saito, 2002 ▶). Ternatins are delphinidin 3-*O*-(6′′-*O*-malonyl)-β-glucoside derivatives that have a 3′,5′-di-*O*-β-glucoside structure in their B-ring, in which both glucosyl residues are alternately acylated and glucosylated in repetitions by *p*-coumaroyl and glucosyl groups (Kazuma *et al.*, 2003 ▶, 2004 ▶). Studies on ternatin biosynthesis in *C. ternatea* revealed that delphinidin is not directly glucosylated at the 3′- or 5′-hydroxyl group, but that glucosylation of delphinidin occurs only when it has a 6′′-*O*-malonyl-β-glucoside at the 3-position. Thus, glucosylation of delphinidin at the 3-hydroxyl group was proposed to be the first key step of ternatin biosynthesis (Kogawa *et al.*, 2007 ▶).

**Figure 1:**
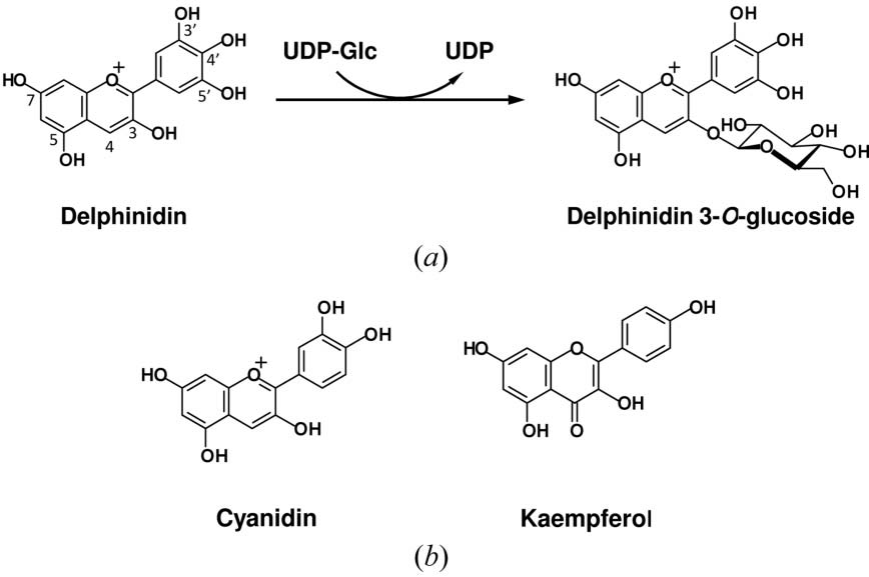
(*a*) Delphinidin conversion to delphinidin 3-*O*-glucoside catalyzed by *Ct*3GT-A. The glucose moiety is transferred from a UDP-Glc donor to the 3-hydroxyl group of delphinidin. (*b*) Chemical structures of cyanidin and kaempferol, the sugar-acceptor substrates of *Vv*GT1.

*Ct*3GT-A was identified in *C. ternatea* as a UDP-glucose:anthocyanidin 3-*O*-glucosyltransferase (GenBank accession No. AB185904) that catalyzes glucosyl transfer from UDP-glucose to anthocyanidins such as delphinidin (Fig. 1*a* ▶). The putative amino acid sequence of *Ct*3GT-A is 45% identical to that of the enzyme *Vv*GT1 from red grape (*Vitis vinifera*), which is a representative uridine diphosphate glycosyltransferase (UGT) with similar acceptor-substrate specificity. *Vv*GT1 is a cyanidin 3-*O*-glycosyltransferase involved in the formation of anthocyanins, with a minor activity toward flavonols such as kaempferol (Fig. 1*b* ▶) (Offen *et al.*, 2006 ▶). The crystal structure of *Vv*GT1, in complex with the non-transferable sugar donor UDP-2-deoxy-2-fluoro glucose (UDP-2FGlc) and the sugar acceptor kaempferol, provided the initial structural basis for understanding the catalytic mechanism and substrate recognition of this enzyme. In addition, the crystal structures of these four plant UGTs have been reported so far: *Medicago truncatula* UGT71G1, a triterpene/flavonoid glycosyltransferase involved in saponin biosynthesis (Shao *et al.*, 2005 ▶), UGT85H2, an (iso)flavonoid glycosyltransferase involved in the biosynthesis of secondary metabolites (Li *et al.*, 2007 ▶), UGT78G1, an (iso)flavonoid glycosyltransferase that functions in anthocyanin biosynthesis (Modolo *et al.*, 2009 ▶) and *Arabidopsis thaliana* UGT72B1, a chloroaniline/chloro­phenol glucosyltransferase in the metabolism of xenobiotics (Brazier-Hicks *et al.*, 2007 ▶). These plant UGTs all have the GT-B fold, one of two general folds found in the UGT superfamily of enzymes (Coutinho *et al.*, 2003 ▶; Breton *et al.*, 2012 ▶), and they possess two N- and C-terminal domains with similar Rossmann-like folds (Wang, 2009 ▶). They also have in common a signature motif known as putative secondary plant glycosyltransferase (PSPG) box near the C-terminus, which is thought to be involved in binding to the UDP moiety of the sugar-donor substrate (Lairson *et al.*, 2008 ▶). However, the relationship between the primary structures of these enzymes and their substrate specificity including regioselective glycosyl­ation remains to be elucidated. Although the crystal structures of several UGTs have been determined, it is still unclear how UGTs distinguish between a large variety of sugar acceptors (*e.g.* anthocyanidins, flavonols and isoflavones) and synthesize many kinds of products.

Here, we present the three-dimensional structure of *Ct*3GT-A determined at a resolution of 1.85 Å by using synchrotron radiation. The structure of *Ct*3GT-A shows the typical GT-B fold conserved in plant UGTs, but structural features of the acceptor-substrate-binding site in *Ct*3GT-A are partly different from those of other UGTs. These findings offer a deep insight into the structure–function relationship of *Ct*3GT-A.

## 2. Materials and methods

### 2.1. Protein expression and purification

The gene encoding *Ct*3GT-A (GenBank accession No. AB185904) was PCR-amplified using the sense primer 5′-GACGACGACAAGATGAAAAACAAGCAGCATG­TTGC-3′ and the antisense primer 5′-GAGGAGAAGCCC­GGTTTAGCTAGAGGAAATCACTTC-3′, and the obtained product was ligated into pET-30 Ek/LIC vector (Novagen). The *Ct*3GT-A cDNA fragment with an enterokinase cleavage site was isolated from the resultant plasmid by digestion with *Bgl*II and *Xho*I, and subcloned into the *Bam*HI/*Sal*I digested pQE31 vector (Qiagen). The recombinant protein was over-expressed in *Escherichia coli* XL1 Blue cells (Stratagene) by adding isopropyl-β-d-galactoside to a final concentration of 1 m*M* and inducing the cells for 20 h at 298 K. The cells were harvested by centrifugation and resuspended in a buffer containing 50 m*M* Tris-HCl (pH 8.0), 500 m*M* NaCl, 20 m*M* imidazole, 1 m*M* dithiothreitol and 0.5 m*M* phenylmethylsulfonyl fluoride. After disrupting the cells by sonication, the cell debris was removed by centrifugation, and the supernatant was applied to a Ni-Sepharose column (GE Healthcare). The eluted fraction containing *Ct*3GT-A was dialyzed against 20 m*M* Tris-HCl (pH 7.4), 200 m*M* NaCl and 2 m*M* CaCl_2_, and the N-terminal His-tag was removed by digestion using recombinant enterokinase (Novagen). Cation-exchange chromatography was carried out next on an SP-5PW column (Tohso, Japan) to purify the enzyme to homogeneity.

### 2.2. Crystallization and data collection

Single crystals of *Ct*3GT-A were obtained using the hanging-drop vapor-diffusion method. After mixing equal volumes of the protein solution (20 mg ml^−1^) and the reservoir solution containing 0.1 *M* sodium citrate tribasic dihydrate (pH 5.6), 0.2 *M* ammonium acetate and 26% (*w*/*v*) polyethylene glycol 4000, the solution was equilibrated against the reservoir solution at 293 K. The crystals, grown up to 0.05 × 0.05 × 0.5 mm in size, were soaked into a cryoprotectant solution containing 25% (*v*/*v*) glycerol in addition to the reservoir solution before measurement.

X-ray diffraction data were collected under a liquid-nitrogen stream (100 K) at beamline BL6A at the Photon Factory (Tsukuba, Japan). The dataset was indexed and processed by *HKL2000* (Otwinowski & Minor, 1997 ▶). The diffraction data statistics are summarized in Table 1 ▶. All graphic images of molecular structure were generated by using the program *PyMOL* (DeLano, 2002 ▶). The atomic coordinates of recombinant wild-type *Ct*3GT-A have been deposited in the RCSB Protein Data Bank (PDB) with the code of 3wc4.

## 3. Results and discussion

### 3.1. Structure determination

The crystals of recombinant wild-type *Ct*3GT-A belong to the space group *P*2_1_, with cell dimensions of *a* = 50.2 Å, *b* = 55.2 Å, *c* = 86.2 Å and β = 105.1°. There was one molecule per crystallographic asymmetric unit with a solvent content of 48% (*v*/*v*) based on a Matthews coefficient (*V*_m_) of 2.4 Å^3^ Da^−1^. The initial phase was solved by molecular replacement using the coordinates of the homologous glycosyltransferase *Vv*GT1 from *V. vinifera* (PDB ID: 2c1z) as a search model.

**Figure 2:**
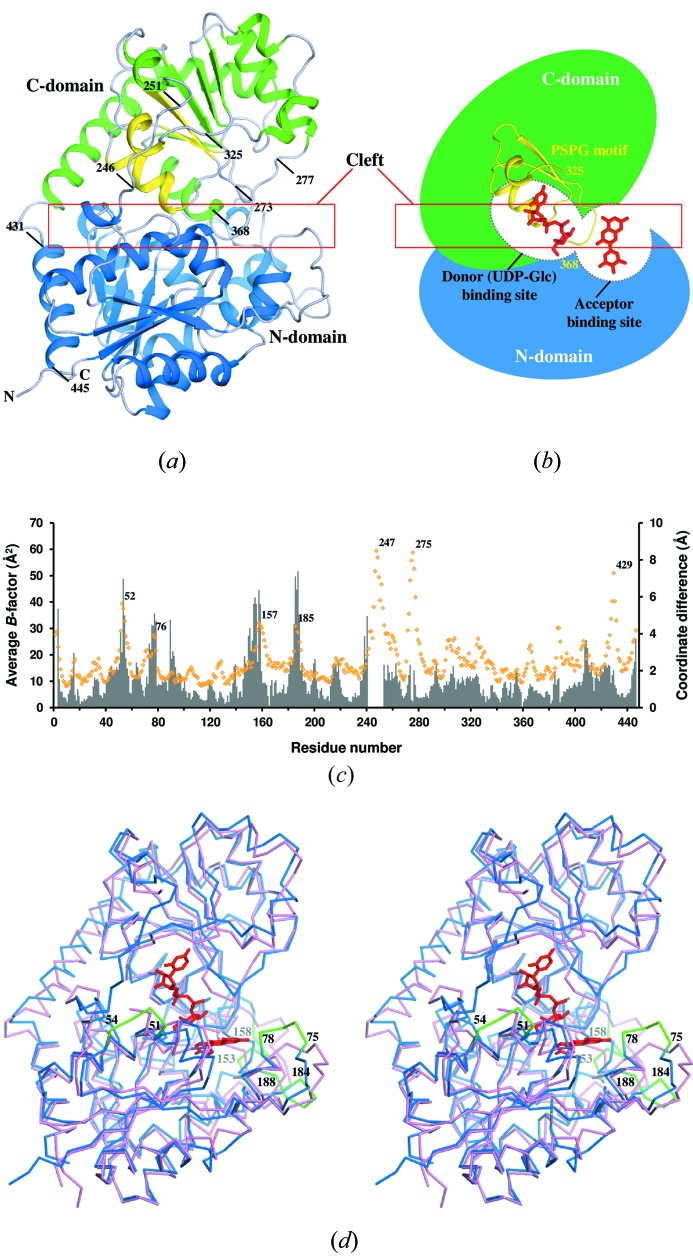
(*a*) Overall structure of recombinant wild-type *Ct*3GT-A. The secondary structures within N-domain and C-domain are colored blue and green, respectively. The PSPG motif from residues 325–368 are colored yellow. The residue numbers indicate the locations of the flexible loop regions and the C-terminal helix associated with the N-domain. (*b*) Schematic representation of the structure of *Ct*3GT-A including the locations of the cleft and the donor- and acceptor-binding sites. The binding site for the donor (UDP-Glc) is formed mainly by the residues from the PSPG motif colored yellow. (*c*) Plots of the *B*-factors for each residue in *Ct*3GT-A and the coordinate differences between *Ct*3GT-A and *Vv*GT1. Average *B*-factor values for the main-chain atoms of *Ct*3GT-A are plotted as orange rhombuses (scale on left-hand axis), with residue numbers denoted on top of the peaks. Coordinate differences between corresponding Cα atoms in the superimposed structures of *Ct*3GT-A and *Vv*GT1 are presented as a bar graph (colored in grey; scale on right-hand axis). Plots corresponding to residues 241–252 in *Ct*3GT-A are missing because of the lack of coordinates in *Vv*GT1. (*d*) Superposition of the structures of *Ct*3GT-A (blue) and *Vv*GT1 (pink; PDB ID: 2c1z). The donor analog UDP-2FGlc and the sugar acceptor kaempferol in the *Vv*GT1 structure are shown as stick models (red). Four loop regions showing significant structural differences are colored green.

An initial model of *Ct*3GT-A was built manually using *COOT* (Emsley & Cowtan, 2004 ▶), and refined subsequently to 1.85 Å resolution with *R*_work_/*R*_free_ of 17.0%/21.1% by using *REFMAC5* in the *CCP4* program suite (Collaborative Computational Project, Number 4, 1994 ▶). All main-chain angles were in the allowed regions of a Ramachandran plot, with 98.4% of the residues in the most-favored regions. The residual electron density that was observed in the protein interior was assumed to be one acetate ion and one glycerol molecule contained in the cryoprotectant solution. The refinement statistics are summarized in Table 1 ▶. The asymmetric unit contained one molecule that corresponds to the physiological monomeric form of *Ct*3GT-A (Fig. 2*a* ▶).

### 3.2. Overall structure of *Ct*3GT-A

*Ct*3GT-A possesses a typical GT-B fold structure comprised of two Rossmann-like β/α/β domains (Fig. 2*a* ▶), which are conserved in plant UGTs (Breton *et al.*, 2012 ▶). The N-terminal β/α/β domain (N-domain) comprising residues 1–244 consists of a seven-stranded twisted parallel β-sheet in the middle surrounded by eight α-helices. The C-terminal β/α/β domain (C-domain) is composed of a twisted β-sheet with six strands accompanied by ten α-helices on its two sides. There is a cleft located between the N- and C-domains (Fig. 2*b* ▶). The cleft was further divided into two cavities that are used as binding sites for the donor (UDP-Glc) and the acceptor substrates (Wang, 2009 ▶). The N- and C-domains are connected by a loop region comprising residues 246–251, which is highly flexible with temperature factors above 41 Å^2^ (Fig. 2*c* ▶). The donor-binding site conserved as a UGT signature ‘PSPG’ motif is located in the C-domain of *Ct*3GT-A, and the C-terminal helix comprising residues 431–445 participates in forming the N-domain after crossing the cleft (Fig. 2*a* ▶).

Structural homology searches performed using the Dali server (Holm & Sander, 1993 ▶) indicated that *Ct*3GT-A was similar to the plant UGTs *Vv*GT1 from *V. vinifera* (PDB ID: 2c1z) and UGT78G1 from *M. truncatula* (PDB ID: 3hbf), with root-mean-square deviations (RMSDs) of 1.9 Å for 432 Cα atoms (Dali *Z*-score of 49.9) and 2.0 Å for 437 Cα atoms (Dali *Z*-score of 48.7), respectively. *Vv*GT1 is an enzyme that preferentially glucosylates cyanidin to yield cyanidin 3-*O*-glucoside in red grape, and its crystal structure has been determined as a Michaelis complex with the non-transferable UDP-2FGlc donor and the flavonol kaempferol (Offen *et al.*, 2006 ▶). UGT78G1 was identified as a multifunctional (iso)flavonoid glycosyltransferase that catalyzes the 3-*O*-glycosylation of formononetin in addition to that of flavonols (Modolo *et al.*, 2009 ▶).

Structural comparison indicated that *Ct*3GT-A and *Vv*GT1 share a common backbone architecture (Fig. 2*d*). The positions of the donor- and acceptor-binding sites in *Vv*GT1 correspond to those of the two cavities in *Ct*3GT-A. The coordinates of UDP-2FGlc and kaempferol in the *Vv*GT1 structure fit well and without any steric hindrance within the cleft of *Ct*3GT-A. When the two enzymes were superimposed using the program *LSQKAB* (CCP4, 1994 ▶), significant displacements (>5 Å) were detected at four loop regions of the N-domain (residues 51–54, 75–78, 153–158 and 184–188) (Fig. 2*c* ▶). Because the loop region containing residues 75–78 is located above the acceptor-binding site, the structural difference may contribute to the differentiation of acceptor-substrate recognition between *Ct*3GT-A and *Vv*GT1.

### 3.3. Structural characteristics for the function of *Ct*3GT-A

**Figure 3:**
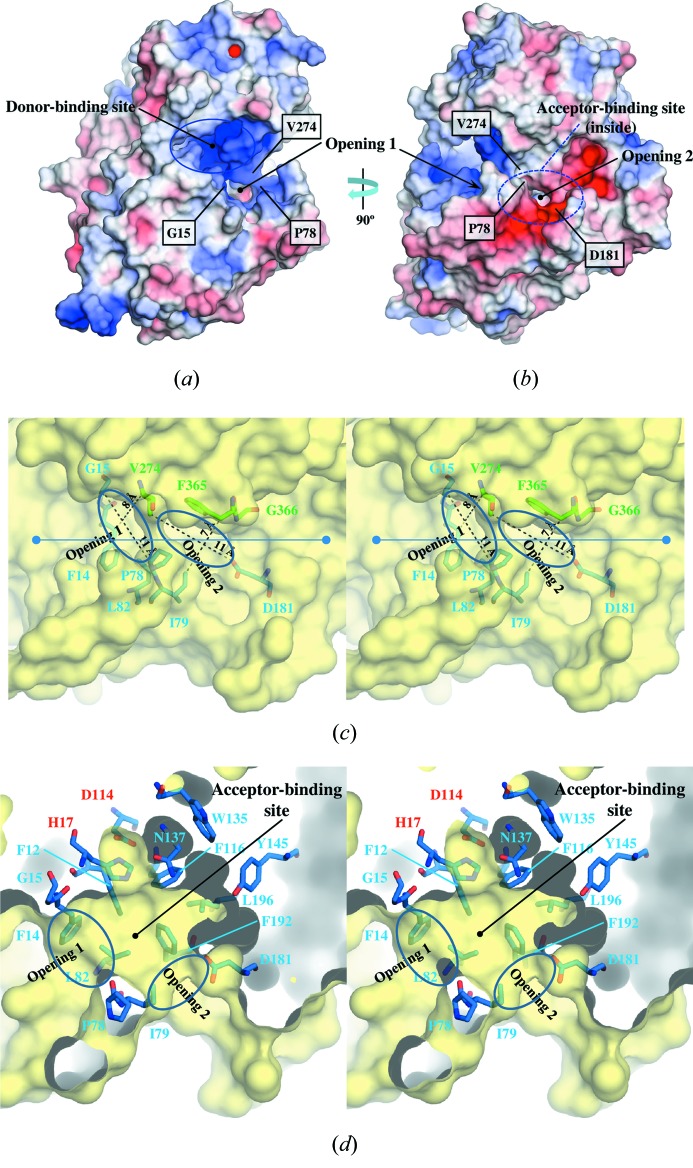
(*a*) Electrostatic surface potential of *Ct*3GT-A viewed from the same direction as in Fig. 2(*a*) ▶. The surfaces are colored by electrostatic potential isocontours from the potential of +5 kT e^−1^ (blue) to −5 kT e^−1^ (red). (*b*) Electrostatic surface potential of *Ct*3GT-A after rotating 90° around the vertical axis. (*c*) Close-up view of the two openings leading to the acceptor-binding site. The residues involved in forming the openings are shown as stick models. The distances showing apparent size of the openings are indicated with dashed lines. (*d*) Cross-section view of the acceptor-binding site after rotating approximately 45° with respect to the figure (along the line) in (*c*). The residues involved in forming the acceptor-binding site are shown as stick models. The conserved catalytic residues, His17 and Asp114, are labeled in red.

To understand the molecular characteristics of *Ct*3GT-A, the electrostatic potential of the protein surface was calculated using *APBS* (Baker *et al.*, 2001 ▶) as shown in Figs. 3(*a*) and 3(*b*) ▶. The donor-binding site located at the surface of *Ct*3GT-A is formed mainly by the residues from the PSPG motif that is highly conserved among plant UGTs and rich in positive charges (Fig. 3*a* ▶). The residues involved in recognizing UDP-2FGlc are almost identical in *Ct*3GT-A and *Vv*GT-1, which is consistent with the fact that these enzymes use the same donor substrate. The donor-binding site is further connected to another cavity for binding acceptor substrates (acceptor-binding site), as shown in Figs. 3(*c*) and 3(*d*) ▶.

The acceptor-binding site is formed mostly by the residues from the N-domain. Besides the hydrophobic residues Phe12, Phe116, Trp135, Tyr145, Phe192 and Leu196, the hydrophilic residues Asn137, Asp181 and Asp367 are arranged to form the acceptor-binding site (Fig. 3*d*). The acceptor-binding site can be accessed from the solvent through two openings, 1 and 2 [Figs. 3(*b*)–3(*d*) ▶], that are separated by the hydrophobic side chains of Pro78 in the N-domain and Val274 in the flexible loop region (residues 273–277) of the C-domain. Opening 1, located near the donor-binding site, is elliptical with a major diameter of 11 Å (between the Cα atoms of Gly15 and Pro78) and a minor diameter of 8 Å (between the Cβ of Phe14 and Cγ2 of Val274), which is formed by hydrophobic residues Phe14, Gly15, Pro78 and Leu82 from the N-domain and V274 from the C-domain (Fig. 3*c* ▶). Opening 2 is formed by the side chains of Ile79, Asp181 and Phe365 and the main chain of Gly366 (Fig. 3*c* ▶), and the size of this elliptical opening is similar to that of opening 1; the major diameter of opening 2 is 11 Å (between the Oδ2 of Asp181 and Cγ1 of Val274) and the minor diameter is 7 Å (between the Cδ1 of Ile79 and Cβ of Phe365). The presence of a hydrophilic residue (Asp181) at opening 2 might help effective passage of the hydrophilic part of the substrate.

The residues His17 and Asp114, located at the bottom of the acceptor-binding site in *Ct*3GT-A, are conserved as the catalytic dyad His20-Asp119 in *Vv*GT1 (Fig. 3*d*), suggesting that *Ct*3GT-A adopts a catalytic mechanism similar to that proposed for *Vv*GT1: the conserved histidine residue acts as a general base to help deprotonation of the 3-hydroxyl group of the acceptor substrate, after which the generated nucleophile attacks the anomeric carbon of the glucose moiety (Breton *et al.*, 2012 ▶). The carboxyl side chain of Asp119 is thought to increase the proton-accepting ability of the imidazole ring as seen in the catalytic mechanism of serine proteases, which have a catalytic triad of Ser-His-Asp with a similar geometry (Wharton, 1998 ▶).

In the acceptor-binding site of *Vv*GT1, the side chains of Ser18, Gln84 and His150 form hydrogen bonds with the flavonol acceptor kaempferol; these residues are substituted with Gly15, Ile79 and Tyr145 in *Ct*3GT-A (Fig. 3*d*). Because the hydrogen bonds with the acceptor substrate are critical for determining molecular orientation within the binding site of *Vv*GT1 (Offen *et al.*, 2006 ▶), the substitutions found in *Ct*3GT-A may enable the differentiation of the acceptor substrate.

Although several crystal structures of acceptor-substrate complexes have been determined, including the structures of flavonol-bound forms of *Vv*GT1 with kaempferol or quercetin and of UGT78G1 bound to myricetin, there is no information for recognition of anthocyanidins, which is presumably due to the instability of anthocyanidins unlike flavonols. Structural studies of *Ct*3GT-A complexes with anthocyanidins are in progress for further understanding the recognition of acceptor substrates in *Ct*3GT-A.

## Supplementary Material

PDB reference: 3wc4

## Figures and Tables

**Table 1:** Data collection and refinement statistics Numbers in parentheses refer to the highest-resolution shell.

Data collection	
X-ray source	PF BL6A
Wavelength (Å)	0.978
Space group	*P*2_1_
Cell dimensions	
*a*, *b*, *c* (Å)	50.2, 55.2, 86.2
β (°)	105.1
Resolution (Å)	1.85 (1.92–1.85)
No. of observed reflections	139758
*R*_merge_	9.9 (42.2)
*I*/σ*I*	37.8 (2.8)
Completeness (%)	99.1 (99.2)
Redundancy	3.6

Refinement	
No. of unique reflections	39179
*R*_work_/*R*_free_	0.170/0.211
No. of atoms	
Protein/water/others	3436/436/16
*B*-factors	
Protein/water/others	18.9/27.1/42.7
RMS deviations	
Bond lengths (Å)	0.013
Bond angles (°)	1.5
PDB code	3wc4
